# Targeting the CaMKII/ERK Interaction in the Heart Prevents Cardiac Hypertrophy

**DOI:** 10.1371/journal.pone.0130477

**Published:** 2015-06-25

**Authors:** Ersilia Cipolletta, Maria Rosaria Rusciano, Angela Serena Maione, Gaetano Santulli, Daniela Sorriento, Carmine Del Giudice, Michele Ciccarelli, Antonietta Franco, Catherine Crola, Pietro Campiglia, Marina Sala, Isabel Gomez-Monterrey, Nicola De Luca, Bruno Trimarco, Guido Iaccarino, Maddalena Illario

**Affiliations:** 1 Department of Medicine and Surgery, University of Salerno, Baronissi (SA), Italy; 2 Department of Translational and Medical Sciences, Federico II University, Naples, Italy; 3 Columbia University Medical Center, College of Physicians & Surgeons, New York Presbyterian Hospital-Manhattan, New York, NY, United States of America; 4 Institute of Biostructure and Bioimaging (IBB) of Italian National Research Council (CNR), Naples, Italy; 5 Department of Advanced Biomedical Science, Federico II University, Naples, Italy; 6 Department of Pharmacy, University of Salerno, Fisciano, Italy; 7 Department of Pharmacy, Federico II University, Naples, Italy; Loyola University Chicago, UNITED STATES

## Abstract

**Aims:**

Activation of Ca^2+^/Calmodulin protein kinase II (CaMKII) is an important step in signaling of cardiac hypertrophy. The molecular mechanisms by which CaMKII integrates with other pathways in the heart are incompletely understood. We hypothesize that CaMKII association with extracellular regulated kinase (ERK), promotes cardiac hypertrophy through ERK nuclear localization.

**Methods and Results:**

In H9C2 cardiomyoblasts, the selective CaMKII peptide inhibitor AntCaNtide, its penetratin conjugated minimal inhibitory sequence analog tat-CN17β, and the MEK/ERK inhibitor UO126 all reduce phenylephrine (PE)-mediated ERK and CaMKII activation and their interaction. Moreover, AntCaNtide or tat-CN17β pretreatment prevented PE induced CaMKII and ERK nuclear accumulation in H9C2s and reduced the hypertrophy responses. To determine the role of CaMKII in cardiac hypertrophy *in vivo*, spontaneously hypertensive rats were subjected to intramyocardial injections of AntCaNtide or tat-CN17β. Left ventricular hypertrophy was evaluated weekly for 3 weeks by cardiac ultrasounds. We observed that the treatment with CaMKII inhibitors induced similar but significant reduction of cardiac size, left ventricular mass, and thickness of cardiac wall. The treatment with CaMKII inhibitors caused a significant reduction of CaMKII and ERK phosphorylation levels and their nuclear localization in the heart.

**Conclusion:**

These results indicate that CaMKII and ERK interact to promote activation in hypertrophy; the inhibition of CaMKII-ERK interaction offers a novel therapeutic approach to limit cardiac hypertrophy.

## Introduction

Calcium/Calmodulin dependent kinase II (CaMKII) belongs to the subfamily of multifunctional Ser/Thr kinases, that phosphorylate a variety of substrates and regulate numerous cellular functions [1–4] that are intimately involved in heart diseases [5–7].

The pathogenetic role of CaMKII in cardiac disorders is confirmed by the up regulation of this kinase in human and animal models of cardiac remodelling and heart failure, [8–11]. Several studies have demonstrated that CaMKII plays important functions in the development of cardiac hypertrophy by activating the impaired gene expression of atrial natriuretic peptide (ANP), brain natriuretic peptide (BNP), beta-myosin heavy chain (β-MHC), and skeletal actin [12]. On the other hand, extracellular signal regulated kinase (ERK) also represent a critical regulator of hypertrophic responses through the phosphorylation of transcription factors and the induction of the expression of hypertrophy-related genes [13]. Thus, activation of the fetal gene program that involves CaMKII and ERK may be regarded as the earliest and most prominent marker of left ventricular hypertrophy (LVH) [14].

Connections between Ca^2+^ signaling and the ERK pathway have been documented in many cell systems [15]. Moreover, we have recently showed that the crosstalk between CaMKII and ERK pathways and their physical interaction regulate the α_1_ Adrenergic Receptors (α_1_AR)-mediated proliferation of vascular smooth muscle cells (VSMC), through their association and we have suggested a model in which CaMKII regulates the Ca^2+^-dependent assembly of the ERK cascade components, and the subcellular localization of both CaMKII and ERK [16]. However, the existence of a mechanistic correlation between the activation of the CaMKII-ERK pathway and the development of cardiac hypertrophy has never been studied.

The implication of CaMKII as a possible mechanism of cardiac disease fosters the search of pharmacological tools to target the kinase in the heart, in order to inhibit maladaptive responses [5, 17–19]. Chemical compounds that possess inhibitory properties on CaMKII are available, including the widely used KN93, which targets the ATP pocket of CaMKs [20]. The poor selectivity of this compound is its major limiting factor for translating the use in clinical setups, due to the elevated risk of undesired effects [21, 22]. The mechanism of inhibition of CaMKII with specific peptides represents an alternative strategy to achieve a selective inhibition of the kinase, based on the sterical inhibition of the conformational change of the kinase needed for its activation [23, 24], rather than with the occupation of the ATP pocket, as for KN93 [21]. A selective peptide inhibitor for CaMKII was derived from the sequence of a natural CaMKII specific protein inhibitor selective to CaMKII, CaM-KIIN [25], which includes a 27-aminoacid sequence (CaM-KNtide: KRPPKLGQIGRSKRVVIEDDRIDDVLK) [26] conjugated with Antennapedia N-terminal domain (KRPPKLGQIGRSKRVVIED) (AntCaNtide), blocks CaMKII-dependent phenotypes *in vitro* [16, 22, 27, 28]. Compared to other pharmacological inhibitors, this peptide has the advantage of CaMKII selectivity over other kinases of the family [26–28]. Recently, we identified the AntCaNtide minimal inhibitory sequence that sits in residues 1–17 (CN17β KRPPKLGQIGRAKRVVI)(27). This novel CN17β peptide recapitulates the inhibitory properties of the parental AntCaNtide peptide. To improve its ability to enter cells, CN17β has been fused with penetrating peptide tat (RKKRRQRRRPPQC). The resulting peptide tat-CN17β retains the inhibitory activity and selectivity for CaMKII [27]. So far there is no evidence of its effectiveness in reducing cardiac myocyte hypertrophy [29]. In this setting also, the use of CaMKII inhibitors can help to understand the molecular elements of the CaMKII-ERK interaction and their functional significance, with the perspective of a novel therapeutic approach to limit pathological cardiac hypertrophy. The aim of this study is therefore to demonstrate in cellular and animal models that the use of CaMKII peptide inhibitors (AntCaNtide and tat-CN17β) is effective to reduce hypertrophy of cardiac myocytes and remodeling of the heart, and identify the mechanism of the crosstalk between the ERK and CaMKII pathways in the hypertrophy phenotype.

## Materials and Methods

### *In vitro* study

#### Cell culture

Cardiomyoblasts H9C2 were purchased from ATCC (CRL-1446) and cultured in Dulbecco’s minimal essential medium (DMEM, GIBCO) supplemented with 10% fetal bovine serum (FBS, GIBCO) 200 mg/mL L-glutamine, 100 units/mL penicillin, and 10 mg/mL streptomycin (Sigma-Aldrich MO.), at 37°C in 0.95 g/L air-0.05 g/L CO_2_. H9C2 cells were studied between passages 4 and 10. To examine the role of CaMKII on cardiac hypertrophy we studied the responses to α_1_AR stimulation, with phenylephrine (PE). H9C2 cells were incubated overnight in DMEM serum-free (FBS 1%) and then exposed to PE (100 nmol/L, Sigma Aldrigh MO.) at different time points. To investigate the effect of CaMKII inhibition on PE-mediated ERK activation, we pretreated H9C2 for 30 min. with the CaMKs inhibitor KN93 (5 μmol/L, methossibenensulphonamide, purchased from Seikagaku); alternatively we used of the selective CaMKII inhibitors AntCaNtide (10 μmol/L) [16, 25, 28] and tat-CN17β (5 μmol/L) [27]. AntCaNtide and tat-CN17β peptides were synthesized and purified at the department of Pharmacy of Salerno as previously described and validated [27]. The penetrating peptide Tat: RKKRRQRRRPPQC (5 μmol/L) was also used as a control in preliminary experiments in which showed no inhibitory activity (data not shown). In order to study the effect of ERK inhibition on PE-mediated CaMKII activation, we pre-treated H9C2s for 30 min. with the MAP Kinase inhibitor UO126 (Promega, WI. 10 μmol/L) [16]. Finally, in another set of experiments, to evaluate the effects of protein Kinase A (PKA) on PE induced CaMKII/ERK interaction, we transfected H9C2s with a plasmid encoding PKA inhibitor single-point mutant gene (PKA-I), a kind gift of Prof. Antonio Feliciello (Federico II University of Naples) [30, 31].

#### Cell Infection and transfection

The catalytically inactive form (rCaMKIIalpha, K42M, impaired ATP binding pocket, (CaMKII DN)) and the wild type (CaMKII-WT, rCaMKIIalpha) variant of CaMKII were subcloned into pSP72 (Promega). Adenoviruses encoding CaMKII catalytically inactive (CaMKII-DN) and wild type (CaMKII-WT) were generated using the AdEasy system (Quantum Biotechnologies) [32–34].

H9C2 cells at ≈ 70% confluence were incubated 1 h at 37°C with 5 mL DMEM containing purified adenovirus at a multiplicity of infection (moi) of 100:1, encoding either the CaMKII-DN, CaMKII-WT variants I or the empty virus as a negative control (Ctr) [16]. 24 h after the infection, the cells were used for the experiments. Transient transfection of the PKA-I plasmid was performed using Lipofectamine 2000 (Invitrogen) in 70% confluent H9C2s.

#### Western Blot and Immunoprecipitation Analysis

To examine the effect of CaMKII inhibition on cardiac hypertrophy, H9C2 cardiomioblasts were stimulated with the α_1_AR agonist, PE (100 nmol/L) after pretreatment with CaMKs inhibitor KN93 (5 μmol/L Seikagaku, Tokyo, Japan) and CaMKII selective inhibitors, AntCaNtide (10 μmol/L) or tat-CN17β (5 μmol/L) for 30 min. At the end of the stimulation, cells were lysed in ice-cold RIPA/SDS buffer [50 mmol/L Tris-HCl (pH 7.5), 150 mmol/L NaCL, 0.01 g/L NP-40, 0.0025 g/L deoxycholate, 2 mmol/L Na_3_VO_4_, 0.2 g/L sodium dodecylsulphate and Protease Inhibtor cocktail (SIGMA)]. Alternatively, left ventricular samples obtained from rats were also lysed in ice-cold RIPA/SDS buffer. Protein concentration was determined using BCA assay kit (Pierce). Endogenous CaMKII was immunoprecipitated with 5 μL of anti-CaMKII antibody and 25 μL of protein A/G plus/protein agarose beads/1 mg total cell extract (Santa Cruz, CA. Code: sc-2003) for three hours at 4°C. Samples were then washed twice with lysis buffer, twice with 1×phosphate-buffered saline, and resuspended in 1×SDS gel loading buffer. The immunoprecipitated kinases were either used to assay activity, or resolved on SDS-PAGE in order to visualize the associated proteins by western blot (WB) and specific antibodies (16). Equal amounts of total cellular extracts or immunocomplexes were electrophoresed on 4–12% SDS-PAGE gel (NOVEX) and transferred to Immobilon P nitrocellulose filter (Millipore Corporation). The membranes were blocked in Tris buffered saline containing 0.002 g/L Tween 20 (TBST) and 0.05 g/L nonfat dry milk. After blocking, the membranes were washed three times in TBST and then incubated overnight at 4°C in TBST containing 5% BSA with primary specific antibody: total ERK1/2 (1:1000; Millipore; Code: 16–283), total CaMKII (1:1000, Santa Cruz Biotechnology, Inc., Heidelberg, Germany Code: sc9035), phospho-tyrosyne p44/p42 ERK (1:1000 Santa Cruz Biotechnology, Inc., Code: sc-16982), Actin (1:1000; Santa Cruz Biotechnology, Inc. Code: sc-58679), Histone 3 (1:1000 Santa Cruz Biotechnology, Inc. Code: sc8655), total CaMKIIα (1:1000, Santa Cruz Biotechnology, Inc Code: sc13141), total CaMKIIβ (1:1000, Life technologies, CA. Code: 13–9800), total CaMKIIγ (1:1000, Santa Cruz Biotechnology, Inc., Code: sc1541), total CaMKIIδ (1:1000, Santa Cruz Biotechnology, Inc., Code: sc5392) and phospho-CaMKII antibody (pT286) (Promega, Madison, WI. Code: V1111). The blots were washed three times in TBST, incubated in appropriate HRP-conjugated secondary antibodies (1:2000, Santa Cruz Biotechnology, Inc.,), dissolved in TBST containing 5% nonfat dry milk and incubated for 1 h at room temperature. After 3 additional washes with TBST, immunoreactive bands were visualized by enhanced chemiluminescence using the ECL-plus detection kit (Amersham Biosciences, UK) and quantified by using ImageQuant software (Amersham Biosciences, UK).

#### Nuclear Extracts Preparation

Nuclei were obtained by cell fraction separation procedure as previously described (16). Briefly, H9C2 were washed in ice-cold phosphate buffer (PBS) and suspended in hypotonic buffer [10 mmol/L Hepes pH 7.9; 10 mmol/L KCl; 0.1 mmol/L EDTA; 0.1 mmol/L EGTA; 0.1 mmol/L NaVO3; 1 mmol/L DTT; 0.5 mmol/L PMSF and Protease Inhibtor cocktail (Sigma Aldrigh. MO)]. Cells were lysed by adding 0.1 g/L Nonidet P-40 and vortexing vigorously. Nuclei were pelleted by centrifugation at 12000 rpm for 30 min. Supernatant (cytosol) was saved for analysis. The nuclei were suspended in hypertonic buffer [20 mmol/L Hepes pH7.9; 400 mmol/L NaCl; 1 mmol/L EDTA; 1 mmol/L EGTA; 0.2 g/L Glycerol; 0.1 mmol/L NaVO3; 1 mmol/L DTT; 0.5 mmol/L PMSF and Protease Inhibitor cocktail (Sigma Aldrigh, MO.)] and rocked 30 min. on a shaking platform at 4°C. The samples were centrifuged at 14000 rpm for 5 min. and the supernatants (nuclear extracts) were saved. Protein concentration was determined by using the Bradford method (Bio-Rad). Cytosol and nuclear extracts were confirmed by WB by using anti-Actin (1:1000 Santa Cruz Biotechnology, Inc. Code: sc8655) and anti-Histone 3 antibodies (1:1000 Santa Cruz Biotechnology, Inc. Code: sc7210).

#### Real Time PCR

Total RNA from H9C2 cell line and isolated ventricular cardiomyocytes, was extracted using Trizol reagent (Invitrogen) and cDNA was synthetized by means of Thermo-Script RT-PCR System (Invitrogen), following the manufacturer instruction. After reverse transcription reaction, real-time quantitative polymerase chain reaction (PCR) was performed with the SYBR Green real time PCR master mix kit (Applied Biosystems, Foster City, CA, USA) as described [35]. The reaction was visualized by SYBR Green Analysis (Applied Biosystem) on StepOne instrument (Applied Biosystem). Primers for gene analysis were as follows:

ANF: For 5’CGTGCCCCGACCCACGCCAGCATGGGCTCC3’; Rev 5’GGCTCCGAGGGCCAGCG-AGCAGAGCCCTCA3’; 18S: For 5’GTAACCCGTTGAACCCATT3’; Rev 5’CCATCCAATCGGTAG-TAGCG3’. CaMKII alpha: For 5’ CCTGTATATCTTGCTGGTTGGG3’; Rev 5’TTGATCAGATCCTT-GGCTTCC3’; CaMKII beta: For 5’TCAAGCCCCAGACAAACAG3’; Rev 5’ TTCCTTAATGCCGT-CCACTG3’. CaMKII gamma: For 5’ AAACCTGTGGATATCTGGGC3’; Rev 5’CTGGTGATGGG-AAATCGTAGG3’. CaMKII delta: For 5’ATAGAAGTTCAAGGCGACCAG3’; Rev 5’ CAGCAAGA-TGTAGAGGATGACG3’. Collagen type I: For 5’GCAACAGTCGATTCACCTACAGCA3’; Rev 5’ AATGTCCAAGGGAGCCACATC3’. Collagen type III: For 5’AGAAGTCTCTGAAGCTGATGG3’; Rev 5’ GCTCCATTCACCAGTTGT3’.Actin: For 5' GGCATCGTGATGGACTCCG 3'; Rew 5' GCTGGAAGGTG-GACAGCGA 3'. All standards and samples were assayed in triplicate. Thermal cycling was initiated with an initial denaturation at 95°C for 5 min. After this initial step, 40 cycles of PCR were performed. Each PCR cycle consisted of heating at 95°C for 15 sec. for melting, 60°C for 30 seconds for annealing and 72°C for 1 min. for the extension. The ratio of fold change was calculated using the Pfaffl method[36].

### *In vivo* study

#### Animals

13-weeks-old normotensive Wistar Kyoto (WKY, n = 11) and spontaneously hypertensive (SHR, n = 32) male rats (Charles River, Calco, LC, Italy) which had access to water and food *ad libitum* were used in these experiments. SHRs were divided into three groups: SHR-AntCaNtide (n = 12) SHR- tat-CN17β (n = 11) and SHR-Control (n = 9). The untreated WKY rats (n = 11) were used as the control. The animals were anesthetized by vaporized isoflurane (4%). After the induction of anesthesia, rats were orotracheally intubated, the inspired concentration of isoflurane was reduced to 2% and lungs were mechanically ventilated (New England Medical Instruments Scientific Inc., Medway, MA, USA) as previously described and validated [35, 37, 38]. The chest was opened under sterile conditions through a right parasternal mini-thoracotomy, to expose the heart. Then, we performed three injections (50 μl each) of AntCaNtide (50 μg/kg diluted in NaCl 0.9% pH 7.4), tat-CN17β (50 μg/kg diluted in NaCl 0.9% pH 7.4) or NaCl 0.9%, as control, into the cardiac wall (anterior, lateral, posterior, apical) [35, 38]. Finally, the chest wall was quickly closed in layers using 3-0 silk suture and animals were observed and monitored until recovery. This procedure was performed once weekly for three consecutive weeks. One week after last treatment, rats were weighed and then euthanized. Hearts were immediately harvested, rinsed 3 times in cold PBS and blotted dry, weighed, divided in left and right ventricles, and then rapidly frozen in liquid nitrogen and stored at -80°C until needed for biochemical studies. Animal procedures were performed in accordance with the guidelines of the Federico II University of Naples Institutional Animal Usage Committee. The investigation conforms to the Guide for the Care and Use of Laboratory Animals published by US National Institute of Health (NIH Publication 85-23, rev. 1996) and approved by the Ethics Committee of the Federico II University.

#### Isolation of ventricular cardiomyocytes for *in vitro* experiments

Adult rat ventricular myocytes were isolated from 12 week old Wistar/Kyoto rat hearts by a standard enzymatic digestion procedure as previously described [39, 40]. Briefly, animals were anesthetized with isofluorane (2% v/v) and 0.5 ml of 100U.I./ml heparin injected (i.p.). After 5 min, chest was opened and heart removed by a transverse cut between aorta and carotid arteries, then cannulated to the perfusion system. Heart was initially perfused (NaCl 120 mmol/L, KCl 14.7 mmol/L, KH2PO4 0.6 mmol/L, Na_2_PO_4_ 0.6 mmol/L, MgSO_4_ 1.2 mmol/L, HEPES 10 mmol/L, Glucose 5.5 mmol/L, butendionemonoxime 10 mmol/L) for 4 min followed by enzymatic digestion with Collagenase type II (Worthington, 1 mg/ml of perfusion buffer) for 12 min. At the end of perfusion, a spongy, flaccid heart was detached from the cannula, atria and right ventricle removed and then cut in small pieces with scissors. Digested prep was filtered and a cell pellet obtained by centrifugation (300 rpm, 2 min.), which has been then resuspended in lysis buffer for western blot.

#### Echocardiography

Transthoracic echocardiography was performed at day 0, 7, 14 and 21 after surgery, using a dedicated small-animal high-resolution ultrasound system (VeVo 770, Visualsonics Inc. Toronto, ON, Canada) equipped with a 17.5 MHz transducer (RMV-716). The rats were anaesthetized by isoflurane (4%) inhalation and maintained by mask ventilation (isoflurane 2%). The chest was shaved by appling a depilatory cream (Veet, Reckitt Benckiser, Milano, Italy) [41]. Left Ventricular (LV) end-diastolic and end-systolic diameters (LVEDD and LVESD, respectively) were measured at the level of the papillary muscles from the parasternal short-axis view as recommended [42]. Intraventricularseptal (IVS) and left ventricular posterior wall thickness (PW) were measured at end diastole. LV fractional shortening (LVFS) was calculated as follow: LVFS = (LVEDD–LVESD)/LVEDD x 100. Left ventricular ejection fraction (LVEF) was calculated using a built-in software of the VeVo 770 [37]. Left ventricular mass (LVM) was calculated according to the following formula, representing the M-mode cubic method: LVM = 1.05×[(IVS+LVEDD+LVPW)3-(LVEDD)3]; LVM was corrected by body weight. All measurements were averaged on 5 consecutive cardiac cycles and analyzed by two experienced investigators blinded to treatment (G.S. and A.A.).

#### Blood Pressure measurement

Blood pressure (BP) was measured as previously described [37, 43]and the record of both systolic (SBP) and diastolic (DBP) was taken using a pressure transducer catheter (Mikro-Tip, Millar Instruments, Inc.; Houston, TX, USA).

#### Histology

Four weeks after AntCaNtide (50 μg/kg), tat-CN17β (50 μg/kg) or NaCl 0.9%, intra-cardiac injection, the hearts were immersion fixed in 10% buffered paraformaldehyde. The tissues were embedded in paraffin, cut at 5 μm, and processed. For Masson trichrome staining of collagen fibers, slides were stained with Weigert Hematoxylin (Sigma-Aldrich, St. Louis, MO) for 10 min., rinsed in PBS (Invitrogen) and then stained with Biebrich scarlet-acid fuchsine (Sigma-Aldrich St. Louis, MO) for 5 min. Slides were rinsed in PBS and stained with phosphomolybdic/phosphotungstic acid solution (Sigma-Aldrich St. Louis, MO) for 5 min. then stained with light green (Sigma-Aldrich) for 5 min. Slides were rinsed in distilled water, dehydrated with 95% and absolute alcohol and a coverslip was placed. For the analysis of cardiomyocytes size, Masson trichrome staining sections were used [37]. The areas (μm^2^) of ~100 cardiac myocytes per heart were measured with the public domain Java image processing program Image by an independent operator blind to the study protocol (DS) [44].

#### Statistical Analysis

One-way ANOVA was performed to compare different groups followed by a Bonferroni post-hoc analysis. Two-way analysis of variance was applied to analyze different parameters among the different groups. A significance level of p<0.05 was assumed for all statistical evaluations. Statistics were computed with dedicated software (GraphPad Prism, San Diego, California). All values in figures are presented as mean±SEM of at least 3 independent experiments. Data from immunoblots were quantified by densitomentric analysis.

## Results

### 1. CaMKII isoforms in cardiac myocytes

**Fig 1:**
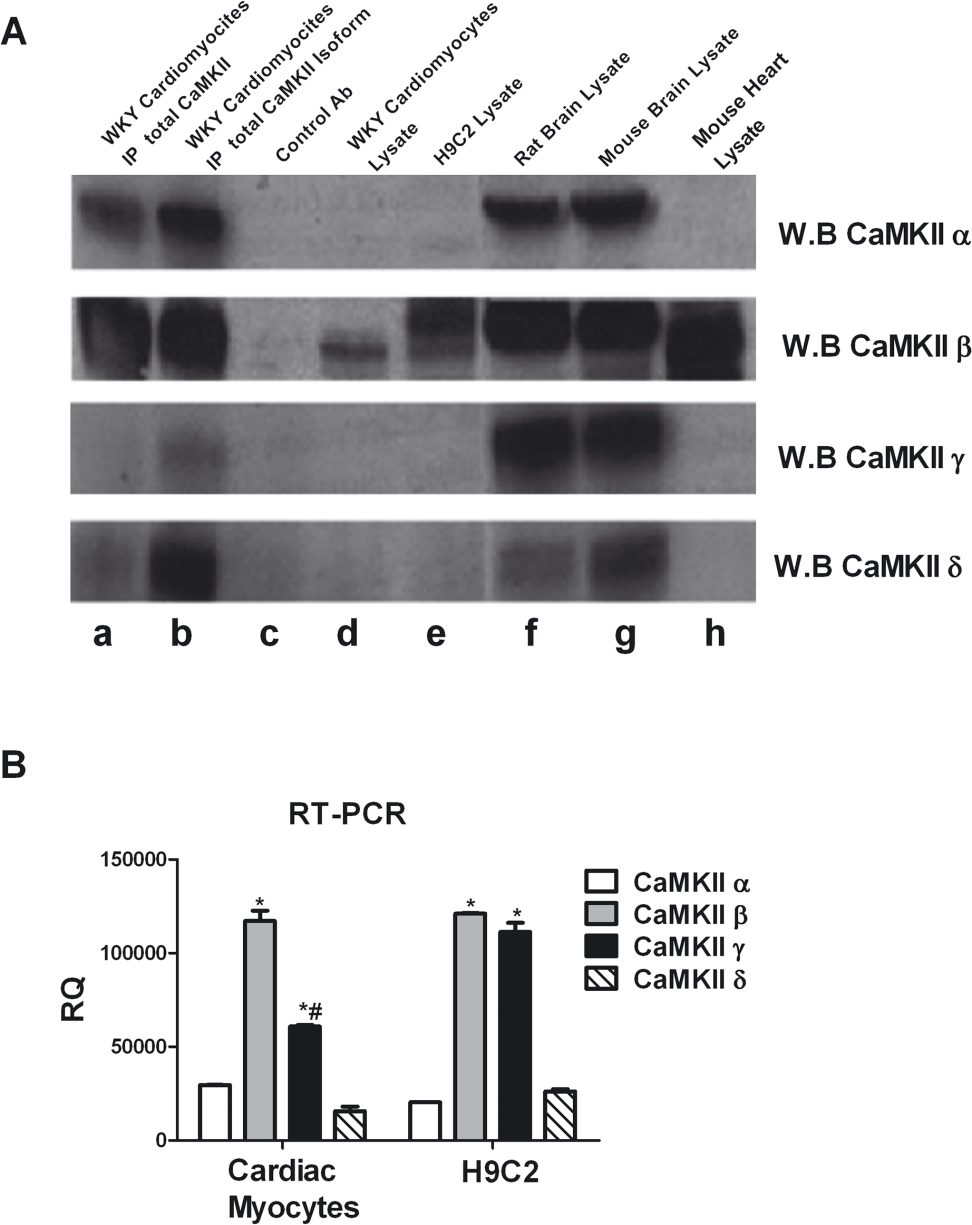
CaMKII isoforms. **A:** To evaluate the expression of CaMKII isoforms in ventricular adult cardiomyocytes from WKY rats, the total cell lysates were immunoprecipitated using anti-CaMKII antibody (lane a) or anti-CaMKII α, β γ or δ specific antibodies (lane b) and together to total samples from WKY cardiomyocytes (lane d) and H9C2 cardiomyoblasts (lane e) were analyzed by western blot with anti-CaMKII α, β γ or δ antibodies as indicated. Total extracts from rat brain (lane f), mouse brain (lane g) and mouse heart (lane h) were used as controls. Total lysates from WKY cardiomyocytes with specific antibody without A/G agarose beads were used as negative control (lane c). **B:** Total RNA from H9C2 cell line and isolated ventricular cardiomyocytes was extracted with standard methods. RT-PCR for CaMKII α, β, γ, and δ was performed as indicated in methods. The representative graph indicates the relative amounts of transcripts for CaMKII isoforms in H9C2s and ventricular adult myocytes from WKY rats. Cycle threshold (Ct) values from 3 independent experiments were normalized to the internal β-actin control. The ratio of fold change was calculated using the Pfaffl method. * = p<0.05 vs CaMKIIα; # = p<0.05 vs CaMKIIβ.

In order to verify the similarity between cultured cardiac myoblast and adult cardiac myocytes, we tested the expression of CaMKII isoforms in H9C2, a cell line of cardiac myoblasts, and ventricular adult myocytes from WKY rats. H9C2 cells express CaMKII α, β and γ isoforms, similar to adult ventricular myocytes. Interestingly, the β isoform is the most abundant, whereas CaMKII α, γ and δ are only detectable after purification by immunoprecipitation from H9C2 cells or rat cardiomyocytes (Fig 1A). These results were confirmed by quantitative RT-PCR analysis (Fig 1B).

### 2. Role of CaMKII-ERK pathways in cardiomyoblasts

**Fig 2:**
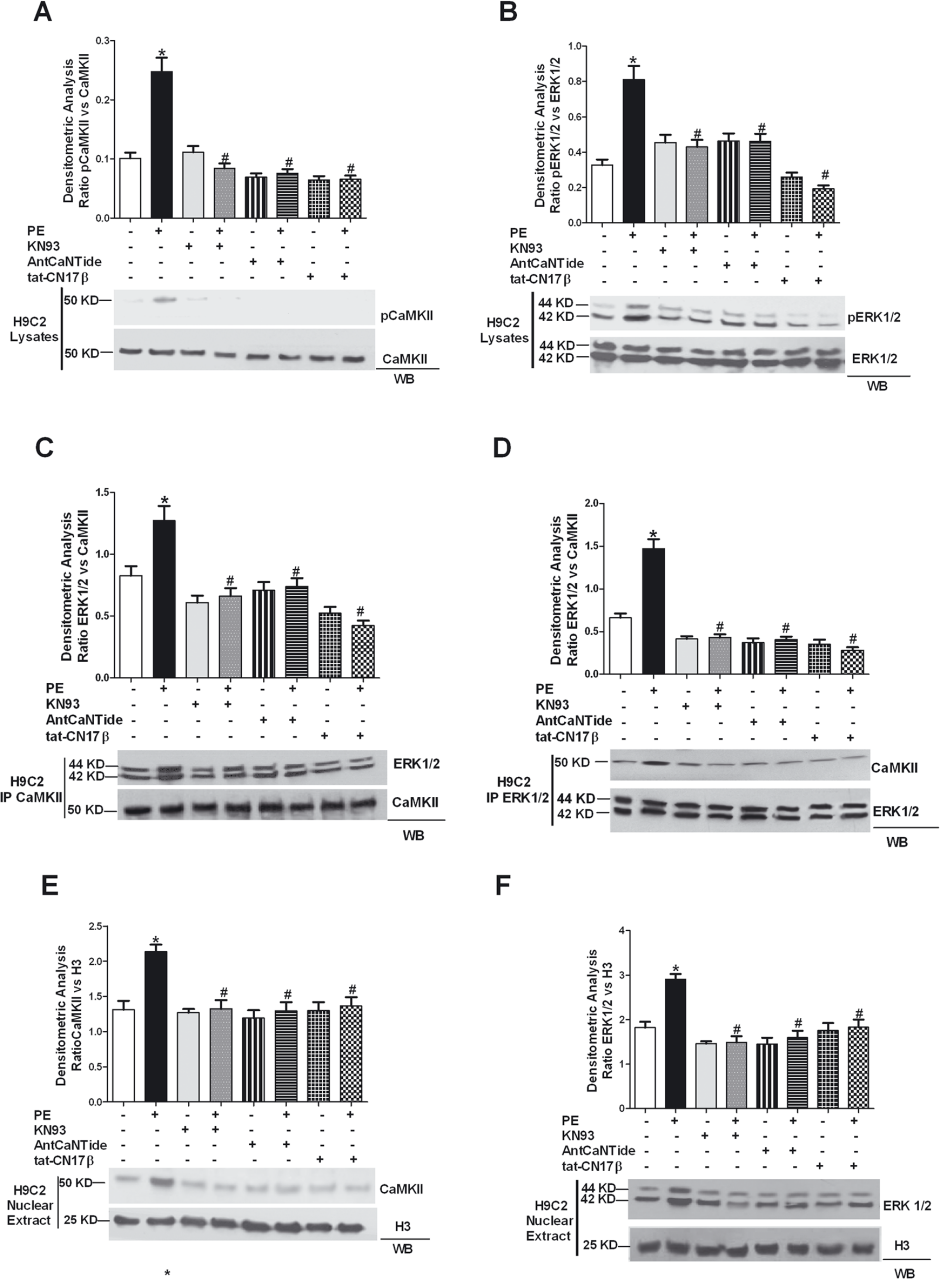
Activation of CaMKII pathway in H9C2 cardiomyoblasts. To investigate the role of the cross talk between CaMKII and ERK on cardiac hypertrophy, we assessed the effect of CaMKII inhibition on PE-induced ERK activation. H9C2s were pretreated with KN93 (5 μmol/L) or AntCaNtide (10 μmol/L) and tat-CN17β (10 μmol/L) for 30 min and then stimulated with PE (100 nmol/L for 15 min.). **A:** H9C2 total cell lysates were analyzed by Western blotting (WB) for phosphothreonine 286 CaMKII (pCaMKII) and total CaMKII (CaMKII), with specific antibodies. Data from immunoblots were quantified by densitometric analysis (DA). pCaMKII levels were corrected by total CaMKII densitometry. * = p<0.05 vs Ctrl; # = p<0.05 vs PE. **B:** Total H9C2 cell extracts were be subjected to WB analysis to visualize phosphotyrosine (pERK1/2) and total ERK (ERK1/2) cell content using anti-pERK1/2 or anti-total ERK1/2 antibodies. pERK1/2 levels were corrected by total ERK1/2 densitometry. * = p<0.05 vs Ctrl; # = p<0.05 vs PE. **C:** H9C2 total lysates were immunoprecipitated using anti-CaMKII antibody. The protein samples underwent WB procedure to visualize ERK and evaluate the association with CaMKII. The experiments were normalized by WB for total CaMKII. ERK1/2 levels were corrected by CaMKII densitometry. * = p<0.05 vs Ctrl; # = p<0.05 vs PE. **D:** To confirm the interaction between CaMKII and ERK in H9C2, total cell lysates were immunoprecipitated using anti-ERK1/2 antibody, and subjected to WB using anti-CAMKII antibody. CaMKII levels were corrected by ERK1/2 densitometry. * = p<0.05 vs Ctrl; # = p<0.05 vs PE. **E:** Nuclear extract from H9C2s were prepared as indicated in the Methods. Nuclear extracts were analyzed by WB for total CaMKII with selective antibody. CaMKII levels were averaged and normalized to histone 3 densitometry. * = p<0.05 vs Ctrl; # = p<0.05 vs PE. **F:** To evaluate nuclear ERK localization, the nuclear extracts were analyzed by WB for total ERK with specific antibody. ERK1/2 levels were normalized to histone 3 densitometry. *, ***P*** < 0.05 ***vs*.** Ctrl; #, ***P*** < 0.05 ***vs*.** PE. Data from all immunoblots were quantified by densitometric analysis. Each data point in all graphs represent the mean±SEM of 3 independent experiments.

To investigate the role of the cross talk between CaMKII and ERK in cardiac hypertrophy, we assessed the effect of CaMKII inhibition on PE-induced ERK activation. We showed that PE activates both CaMKII and ERK in H9C2s and pretreatment with the CaMK inhibitor KN93, as well as with CaMKII-specific inhibitors (*i*.*e*. the cell-permeant peptide, AntCaNtide or the AntCaNtide minimal inhibitory sequence tat-CN17β) inhibits CaMK and also ERK phosphorylation (Fig 2A and 2B). Furthermore, PE induces CaMKII-dependent association with ERK, as shown by co-immunoprecipitation assays. CaMKII/ERK interaction is prevented by CaMKII inhibition with KN93, AntCaNtide or tat-CN17β (Fig 2C and 2D).

**Fig 3:**
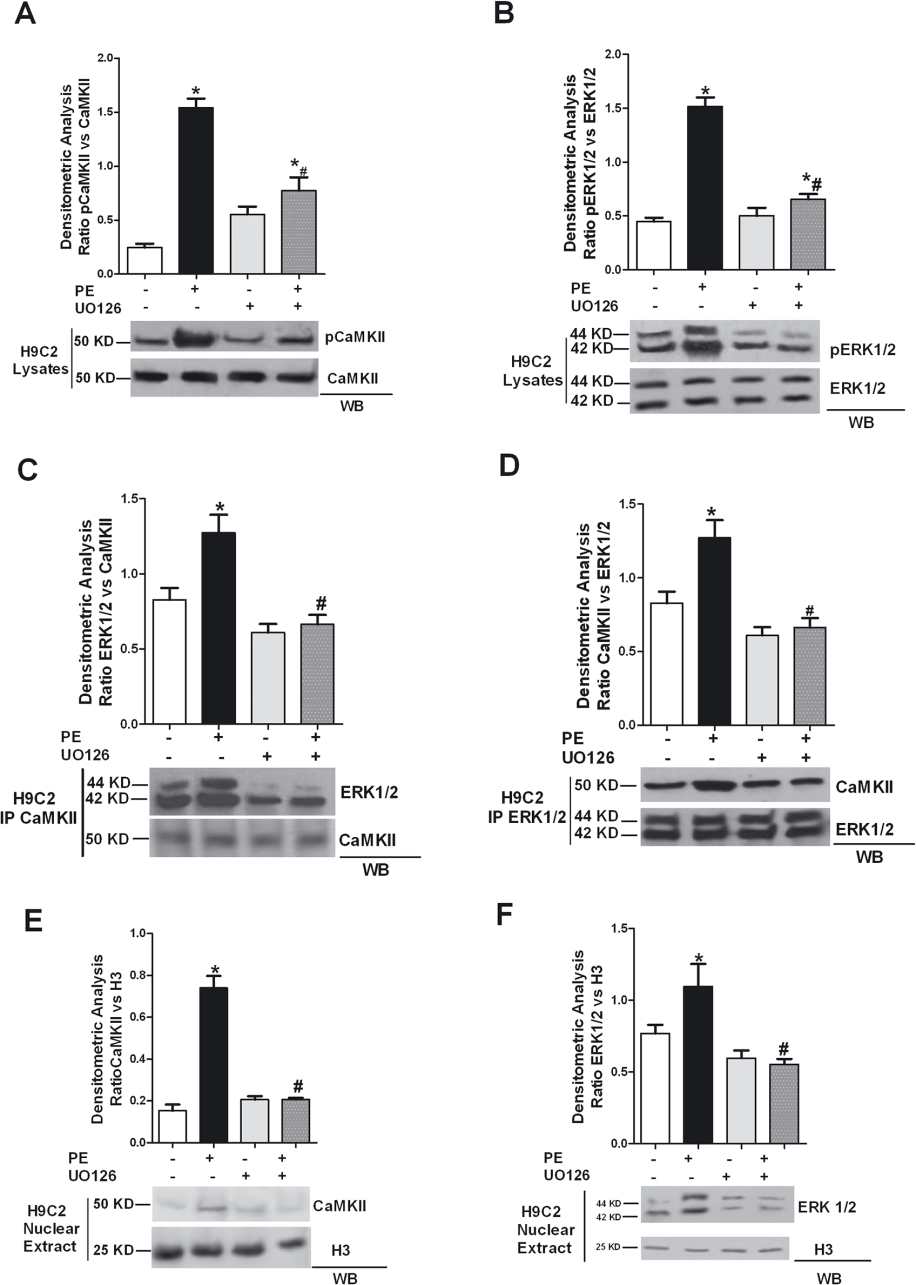
Inhibition of ERK pathway downregulates PE-induced CaMKII activation in H9C2 cardiomyoblasts. **A:** H9C2 cardiomyoblasts were exposed to UO126 (10 μmol/L) for 30 min., and then stimulated with 100 nM phenylephrine (PE) for 15 min. Total cell extracts were analyzed by western blot for phosphothreonine 286 CaMKII (pCaMKII) and CaMKII with specific antibodies. pCaMKII levels were corrected by CaMKII densitometry. *, *P* < 0.05 *vs*. Ctrl; #, *P* < 0.05 *vs*. PE. **B:** H9C2s were stimulated with PE after pretreatment with UO126 (10 μmol/L for 30 min.). Total lisates were analyzed by WB for pERK with specific antibody. pERK1/2 levels were corrected by ERK1/2 densitometry. * = p<0.05 vs Ctrl; # = p<0.05 vs PE. **C:** H9C2s were stimulated with 100 nmol/L PE for 15 minutes following 30 min. pretreatment with UO 126 (10 μmol/L). CaMKII was immunoprecipitated from cell lysates using a specific anti- CaMKII antibody, and ERK (ERK1/2) was visualized by WB to evaluate its association with CaMKII. ERK1/2 levels were corrected by CaMKII densitometry. * = p<0.05 vs Ctrl;# = p<0.05 vs PE. **D:** To confirm the interaction between CaMKII and ERK in H9C2s, total cell lysates were immunoprecipitated using anti-ERK1/2 antibody, and subjected to western blot using anti-CAMKII antibody. CaMKII levels were corrected by ERK1/2 densitometry.* = p<0.05 vs Ctrl; # = p<0.05 vs PE. **E:** After pharmacological inhibition of ERK and stimulation with PE, nuclear extract from H9C2s were prepared as indicated in the methods section. Nuclear extracts were analyzed by WB for total CaMKII with specific antibody. CaMKII levels were averaged and normalized to histone 3 densitometry. *, ***P*** < 0.05 ***vs*.** Ctrl; # = p<0.05 vs PE. **F:** To confirm the effects PE induced ERK nuclear localization after pretreatment with UO126, nuclear extracts were analyzed by WB for total ERK1/2 with specific antibody. ERK1/2 levels were normalized to histone 3 densitometry. *, ***P*** < 0.05 ***vs*.** Ctrl; # = p<0.05 vs PE. Data from all immunoblots were quantified by densitometric analysis. Each data point in all graphs represents the mean±SEM of 3 independent experiments.

**Fig 4:**
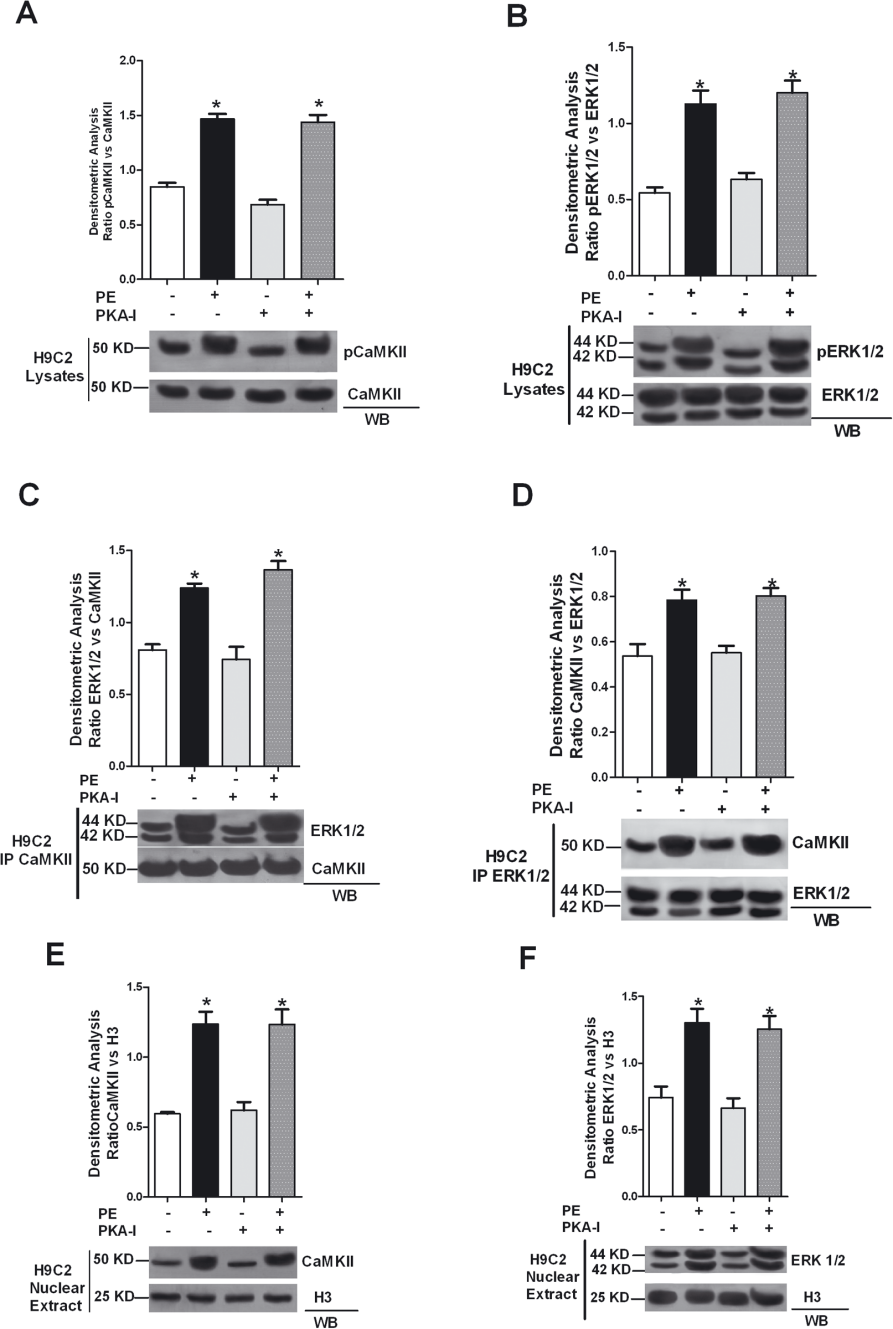
PKA inhibition does not modify PE-induced CaMKII/ERK activation in H9C2 cardiomyoblasts. **A:** H9C2 cardiomyoblasts were transfected with PKA-I and then stimulated with 100 nm phenylephrine (PE) for 15 min. Total cell extracts were analyzed by western blot for phosphothreonine 286 CaMKII (pCaMKII) and CaMKII with specific antibodies. pCaMKII levels were corrected by CaMKII densitometry. *, *P* < 0.05 *vs*. Ctrl. **B:** H9C2s were stimulated with PE after transfection with PKA-I. Total lysates were analysed by WB for pERK with specific antibody. pERK1/2 levels were corrected by ERK1/2 densitometry. * = p<0.05 vs Ctrl. **C:** To evaluate that PKA inhibition does not modify CaMKII/ERK interaction, H9C2s were stimulated with 100 nmol/L PE for 15 minutes following 30 min. pretreatment with PKA-I (10 μmol/L). CaMKII was immunoprecipitated from cell lysates using a specific anti- CaMKII antibody, and ERK1/2 was visualized by WB to evaluate its association with CaMKII. ERK1/2 levels were corrected by CaMKII densitometry. * = p<0.05 vs Ctrl. **D:** To confirm the interaction between CaMKII and ERK in H9C2s, total cell lysates were immunoprecipitated using anti-ERK1/2 antibody, and subjected to western blot using anti-CAMKII antibody. CaMKII levels were corrected by ERK1/2 densitometry. * = p<0.05 vs Ctrl. **E:** H9C2s were stimulated with PE after trasfection with PKA-I. Subsequently, nuclear extract were prepared from the cells as indicated in the methods section. Nuclear extracts were analyzed by WB for total CaMKII with specific antibody. CaMKII levels were averaged and normalized to histone 3 densitometry. *, ***P*** < 0.05 ***vs*.** Ctrl. **F:** To confirm that PE induced ERK nuclear localization was independent from PKA inhibition, H9C2s were transfected with PKA-I and then stimulated with PE. Nuclear extracts were analyzed by WB for total ERK1/2 with specific antibody. ERK1/2 levels were normalized to histone 3 densitometry. *, ***P*** < 0.05 ***vs*.** Ctrl. Data from all immunoblots were quantified by densitometric analysis. Each data point in all graphs represents the mean±SEM of 3 independent experiments.

Next, we evaluated the intracellular localization of ERK and CaMKII to study whether CaMKII inhibition affects the subcellular compartmentalization of one or both kinases. PE stimulation induces both CAMKII and ERK nuclear accumulation; KN93, AntCaNtide and tat-CN17β prevent PE induced nuclear localization of both kinases (Fig 2E and 2F). Previously, we showed in VSMC cells that the interaction between CaMKII and ERK resulted in a significant increase of autonomous CaMKII activity and we demonstrated that ERK activity is required to interact with and activate CaMKII (16). Interestingly, the inhibition of ERK by UO126 in turn reduces CaMK2 phosphorylation also in cardiomyoblasts (Fig 3A and 3B), inhibits the interaction of both CaMKII and ERK (Fig 3C and 3D), as well as PE-induced nuclear localization of ERK and CaMKII (Fig 3E and 3F). It is possible to speculate that the effect of PE is mediated by a non-selective activation of the βAdrenergic Receptor and consequent PKA activity. To rule out this hypothesis, we transfected H9C2 with the PKA-I plasmid to inhibit PKA and verified under these conditions PE induced CaMKII/ERK interaction induced by PE. PKA-I did not impair PE-induced CaMKII or ERK phosphorylation (Fig 4A and 4B) nor did we observe inhibition of PE mediated CaMKII/ERK interaction (Fig 4C and 4D) or PE-induced nuclear localization of either kinases (Fig 4E and 4F). Altogether, our results demonstrate CaMKII-dependent ERK activation after PE stimulation and support the concept that ERK and CaMKII transactivate reciprocally.

### 3. CaMKII-dependent regulation of the hypertrophy marker Atrial Natriuretic Factor

**Fig 5:**
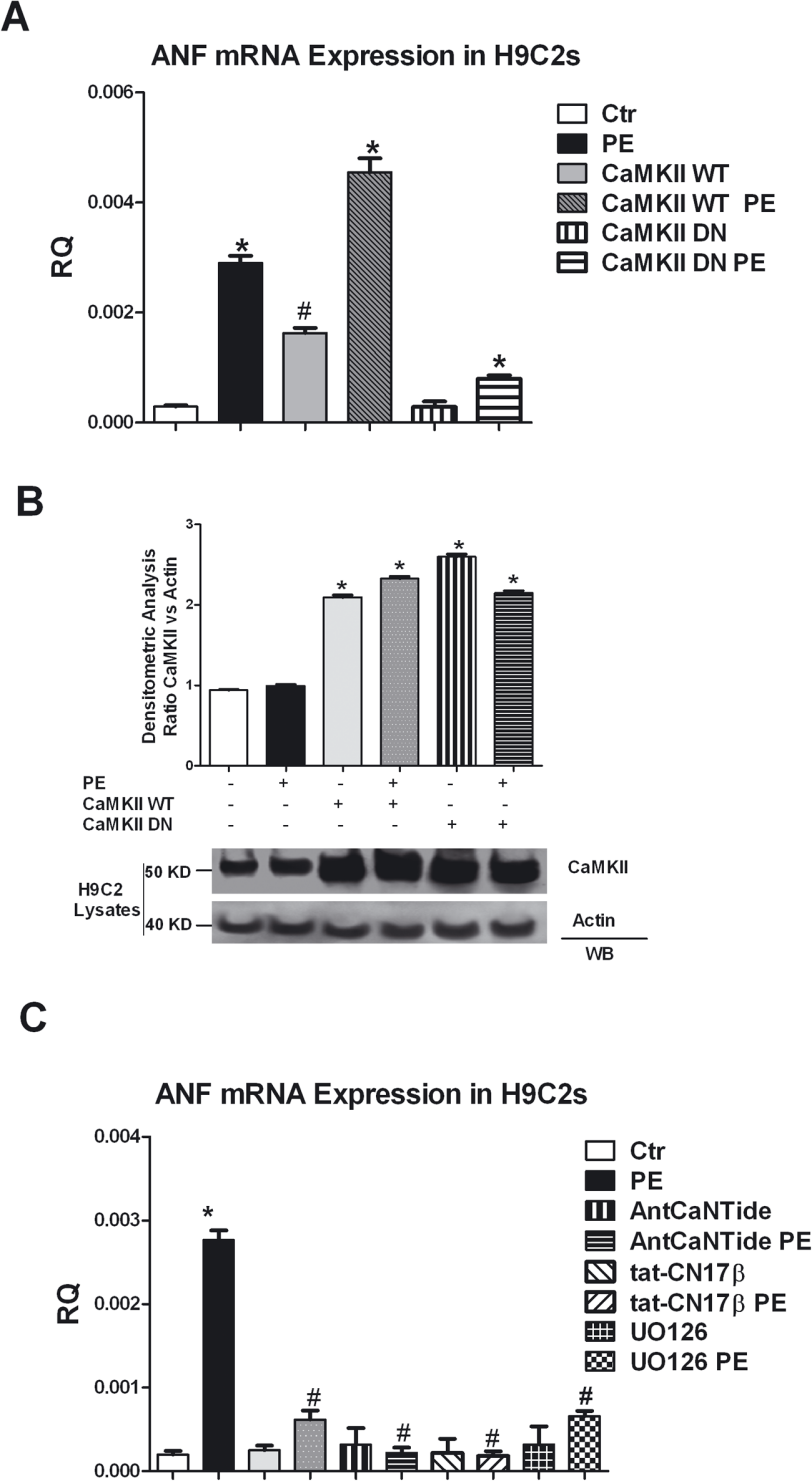
CaMKII/ERK-dependent regulation of the hypertrophy marker ANF. **A:** H9C2 cells at ≈ 70% confluence were incubated 1 h at 37°C with 5 mL DMEM containing purified adenovirus at a multiplicity of infection (moi) of 100:1, encoding either the kinase-dead (CaMKII-DN, rCaMKIIdelta, K42M), or the wild type (CaMKII-WT, rCaMKIIdelta) variant of CaMKII or the empty virus as a negative control (Ctr). 48 h after the infection, the cells were stimulated with PE 100 nM for 24 h. Total RNA was isolated from H9C2s using TRIzol reagent, and cDNA was synthesized by means of a Thermo-Script RT-PCR System, following the manufacturer’s instruction. Then ANP gene expression was evaluated by real-time PCR. Results are expressed as mean±SEM from 3 independent experiments. The ratio of fold change was calculated using the Pfaffl method[36]. * = p<0.05 vs Ctrl; # = p<0.05 vs PE. **B:** The H9C2s infected with adenoviruses encoding wilde type CaMKII (CaMKII-WT) and kinase dead CaMKII (CaMKII-DN) were stimulated with PE 100 nM for 24 h. Total cell lisates were analyzed by WB for total CaMKII with specific antibody. CaMKII levels were corrected by Actin densitometry. Data from the immunoblots were quantified by densitometric analysis.* = p<0.05 vs Ctrl. Each data point in all graphs represents the mean±SEM of 3 independent experiments. **C:** H9C2 cells were pretreated with CaMK inhibitor KN93 (5 μmol/L), the selective inhibitors AntCaNtide (10 μmol/L) and tat-CN17β (10 μmol/L) and ERK specific inhibitor pathway UO126 (10 μmol/L) for 30 min. and then stimulated with PE (100 nmol/L) for 24 h. cDNA was synthesized from RNA obtained from H9C2s as indicated above. The ANF gene expression was evaluated by real-time PCR. Results are expressed as mean±SEM from 3 independent experiments. The ratio of fold change was calculated using the Pfaffl method[36]. * = p<0.05 vs Ctrl; # = p<0.05 vs PE.

The atrial natriuretic peptide (ANP) is an established marker of cardiac hypertrophy, and PE a widely recognized inducer of cardiac hypertrophy [45], causes ANP expression in H9C2 cells. To demonstrate the role of CaMKII in cardiac hypertrophy, H9C2 cardiomioblasts were incubated with adenoviral constructs encoding CaMKII catalytically inactive (CaMKII-DN), CaMKII wild-type (CaMKII-WT) or the empty virus as a negative control and then stimulated with PE for 24 h to assess ANP expression levels. The overexpression of CaMKII-WT determines an increase of the expression levels of ANP both in basal conditions and after stimulation with PE compared to controls. On the other hand, CaMKII-DN infection significantly reduces ANP levels (Fig 5A). To confirm the expression of CaMKII-WT and CaMKII-DN after infection in H9C2s, the total cell lysates were subjected to western blot analysis for CaMKII (Fig 5B). Finally, the efficacy of CaMKII inhibitors to prevent PE induced hypertrophic responses in H9C2s, is confirmed by the use of KN93, AntCaNtide and tat-CN17β, which all significantly reduced the levels of ANP expression induced by PE (Fig 5C). A similar result is obtained by the inhibition of ERK pathway with UO126 (Fig 5C). These data are consistent with the crosstalk between CaMKII and ERK pathways, and suggest the existence of a correlation between the activation of CaMKII-ERK pathway and ANP activation.

### 4. CaMKII selective inhibition with specific peptides blocks cardiac hypertrophy in spontaneously hypertensive rats

**Fig 6:**
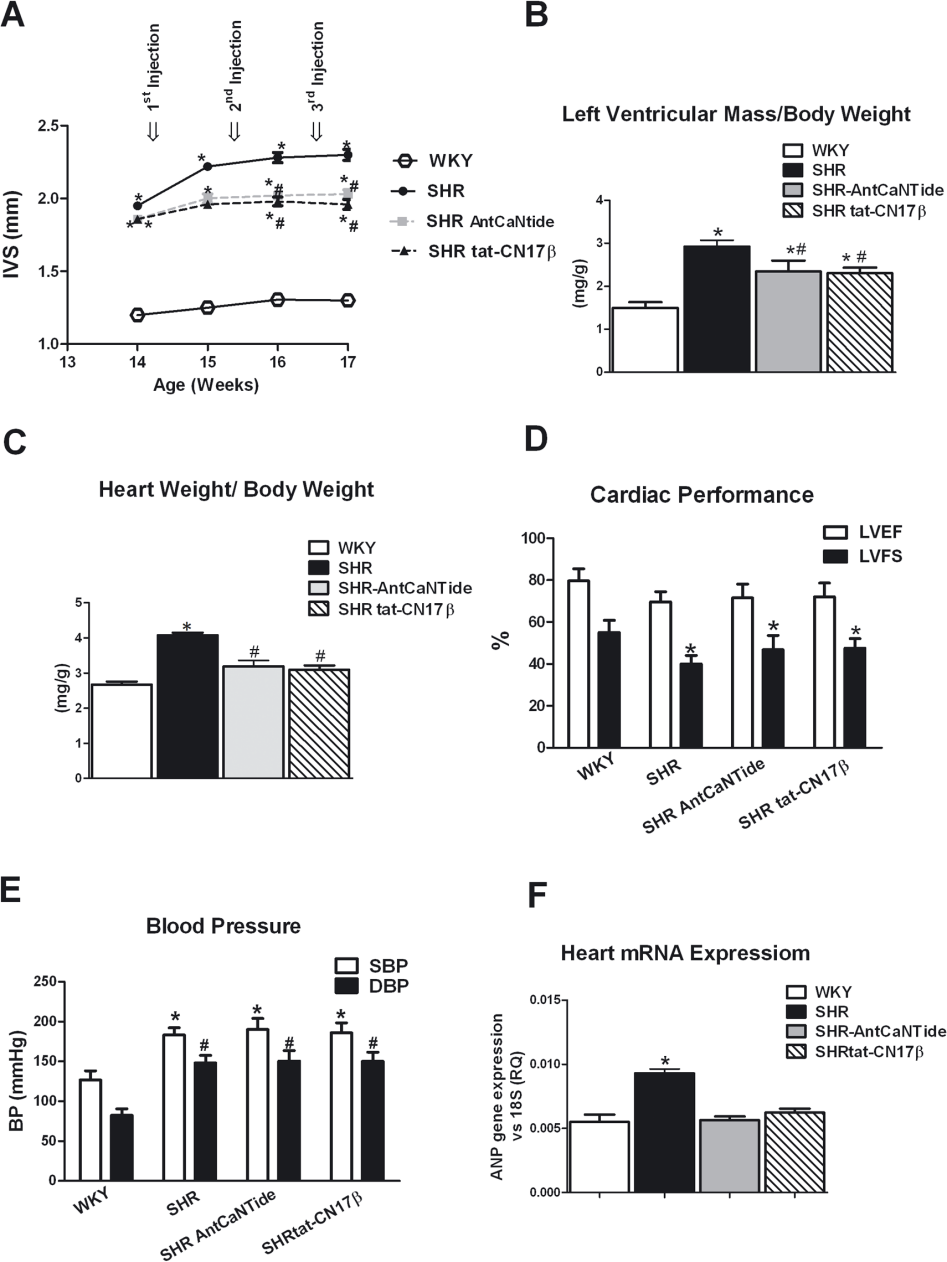
The effect of CaMKII selective peptide inhibitors in vivo in SHRs. **A:** SHRs were subjected to intramural cardiac injections at the age of 13 week and repeated after 7 and 14 days. Transthoracic echocardiography was performed at each drug administration and once more at the day of euthanasia in untreated WKY rats, AntCaNtide SHRs, tat-CN17β SHRs, and control SHRs (SHR) using a dedicated small-animal high-resolution-ultrasound system (Vevo 770, VisualSonics). ANTCaNtide-SHRs and tat-CN17β-SHRs caused reduction of interventricularseptal (IVS) thickness compared to SHR (*P<0.05 vs WKY; #*P*<0.05 vs SHR). **B, C:** At the end of treatment, rats were weighed and then euthanized. Hearts were immediately removed, rinsed 3 times in cold PBS and blotted dry, weighed and then rapidly frozen. The HW/BW ratio (**B**) and LVM/BW ratio (**C**) were measured in ANTCaNtide-SHRs and tat-CN17β-SHRs and compared to WKY and SHR hearts (*P<0.05 vs WKY; #*P*<0.05 vs SHR). **D:** Cardiac Performance at the end of the treatment was assessed by ultrasound. All measurements were averaged on 5 consecutive cardiac cycles and analysed by two experienced investigators blinded to treatment (GS, MC). No differences were observed in LVFS and LVEF in ANTCaNtide and tat-CN17β SHRs when compared with SHR (*P<0.05 vs WKY). **E:** To evaluate the involvement of BP values in CaMKII–dependent regulation of cardiac hypertrophy, SBP and DBP values were assessed in AntCaNtide- and tat-CN17β- treated and control. The pressure values ​​in the graph represent the measurements made at the end of treatment (*P<0.05 vs WKY-SBP; #*P*<0.05 vs WKY-DBP). **F:** At the end of the treatment the total RNA was isolated from myocardial sample using TRIzol reagent, and cDNA was synthesized by means of a Thermo-Script RT-PCR System, following the manufacturer’s instruction. ANP gene expression was evaluated by real-time PCR in AntCaNtide- and tat-CN17β-SHR rats compared with WKY and SHR (*P<0.05 vs WKY). Results are expressed as mean±SEM from 3 independent experiments. The ratio of fold change was calculated using the Pfaffl method[36].

To transpose in *in vivo* model the effect of CaMKII inhibition, we used SHR, an animal model of hypertension-induced left ventricular hypertrophy (LVH). AntCantide, tat-CN17β or a saline solution were injected in the cardiac wall of the SHRs at a time point when LVH develops. LVH was evaluated weekly for 3 weeks by echocardiography. Both AntCaNtide and tat-CN17β treatments efficiently reduced cardiac wall thickness (Fig 6A) and LVM (Fig 6B). Accordingly, at the end of the observation period, the heart weight (HW) to the body weight ratio showed a significant reduction (Fig 6C). A similar beneficial effect could be observed on left ventricle function, assessed by ultrasound analysis. Indeed, compared to normotensive WKY rats, LV ejection fraction (LVEF) and LV fractional shortening (LVFS), were depressed in the SHR control group (Fig 6D). The treatment with either AntCantide or tat-CN17β tends to improve these parameters without however reaching statistical significance (Fig 6D). To rule out changes in hemodynamics as a possible mechanism of regulation of LVH, we measured blood pressure in rats after the treatment and verified that there were no significant effects on this parameter (Fig 6E). Biochemical analysis confirmed the reduction of LVH related biochemical responses induced by AntCantide and tat-CN17β, as assessed by the expression of the hypertrophy marker gene ANP (Fig 6F). Because increased cell size and augmented interstitial fibrosis are key processes in the progression of pathological cardiac remodeling, we performed Masson trichrome staining to assess the effects of AntCantide and tat-CN17β treatment on SHR hearts. Intramyocardial injection of both CaMKII inhibitors significantly attenuated the amount of fibrosis such as cardiomyocytes size when compared with SHR controls.

**Fig 7:**
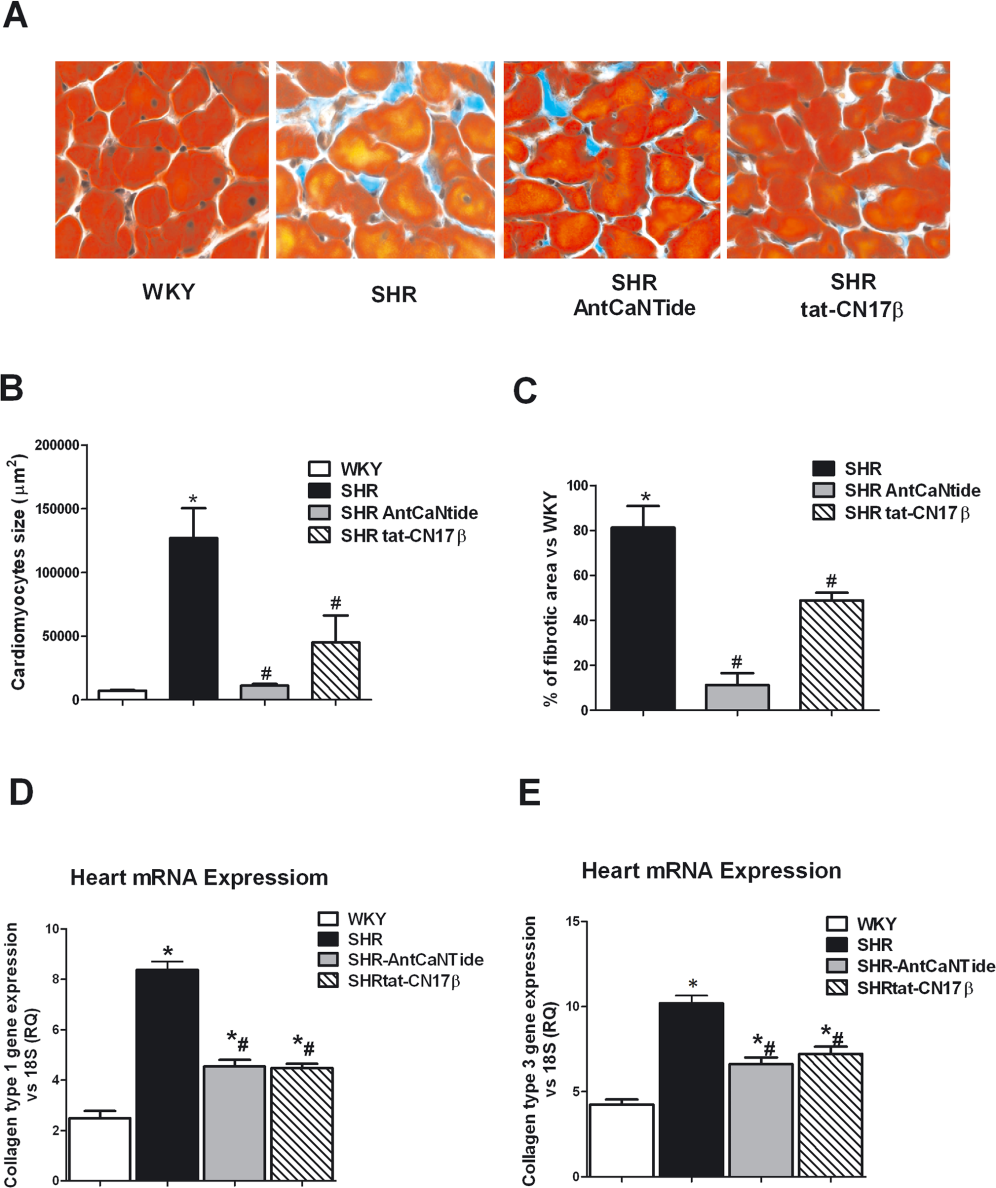
CaMKII selective peptide inhibitors reduces myocardial fibrosis in SHRs. **A:** Paraffin-blocked tissues from left ventricle were sectioned and stained with Masson's trichrome after three weeks of CaMKII selective inhibitors (×20). SHR treated with AntCaNtide and tat-CN17β showed a decrease of interstitial fibrosis and reduced cardiomyocytes size when compared to SHR untreated group. The section stained with Masson's trichrome of WKY was used as control. **B:** Cardiomyocytes size was measured by Image J software, and means of areas are showed in the histogram ((*P<0.05 vs WKY; # P<0.05 vs SHR). Images are representative of 3 independent experiments (magnification ×60; black bar = 100 μm). **C:** Quantification of fibrosis was done by Image J software, and percent of fibrotic areas compared to WKY are shown in the histogram (*P<0.05 vs WKY). Images are representative of 3 independent experiments. **D, E:** To confirm that intramyocardial injection with CAMKII inhibitors AntCaNtide and tat-CN17β blunted interstitial fibrosis we have evaluated mRNA levels of fibrosis biomarkers such as collagen type I (**D**) and collagen type III (**E**) using RT-PCR. *P<0.05 vs WKY; # *P*<0.05 vs SHR. Results are the mean of 3 independent experiments.

Histological analysis of *SHR* hearts showed common elements of hypertension-induced LVH, including increased cell size and augmented interstitial fibrosis compared to WKY (Fig 7A). The improvement of both parameters was confirmed by digital measurements (Fig 7B and 7C). To further confirm that the treatment with CaMKII selective inhibitors reduced interstitial fibrosis of SHR hearts, we evaluated the collagen expression. The quantitative real-time PCR demonstrated that the transcript levels of both Collagen I and Collagen III were significantly lower in SHR hearts treated with with AntCantide or tat-CN17β when compared to SHR controls (Fig 7D).

### 5. The selective inhibition of CaMKII regulates CaMKII/ERK subcellular localization in left ventricles of Spontaneously Hypertensive rats

**Fig 8:**
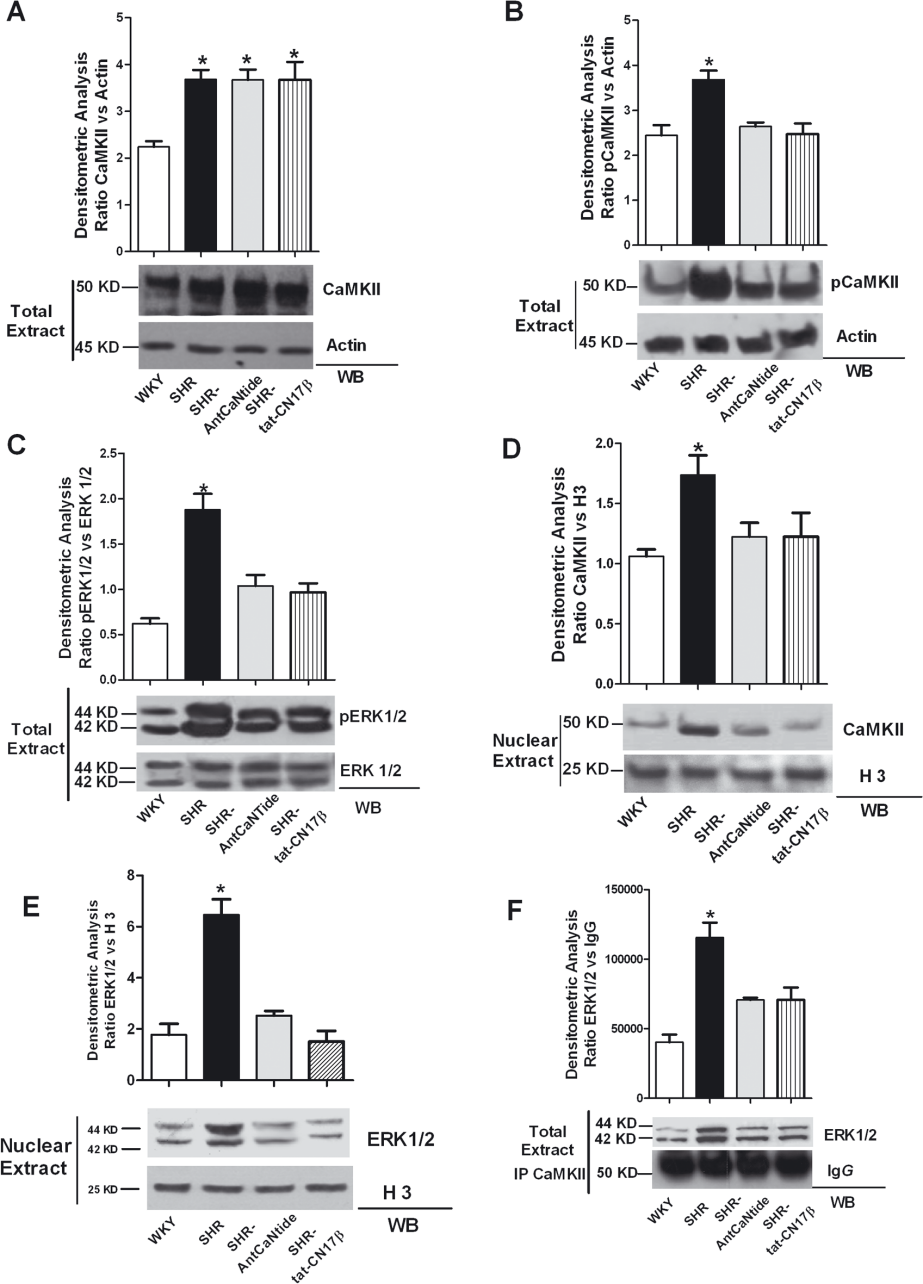
Effects of CaMKII inhibition on CaMKII/ERK pathway *in vivo* in SHR. **A**: After three weeks of treatments, heart were harvested, weighted, and samples from WKY, SHR- AntCaNtide, SHR- tat-CN17β, and SHR-Control total lysates were prepared from the left ventricular samples. Whole lysates were subjected western blotting analysis with anti‐CAMKII antibody. CAMKII levels were corrected by Actin densitometry.* = p<0.05 vs WKY. **B**: To assess CAMKII phosphorylation levels in LV after CaMKII inhibitors pretreatment, total lysate samples from WKY, SHR-AntCaNtide, SHR- tat-CN17β, and SHR-Control were analyzed by WB for anti-phosphothreonine 286 CaMKII antibody (pCaMKII). pCaMKII levels were corrected by Actin densitometry.* = p<0.01 vs WKY. **C:** Total cell extracts of LV from WKY, SHR-AntCaNtide, SHR- tat-CN17β, and SHR-Control were analyzed by WB with anti-pERK (pERK1/2) or anti- total ERK1/2. pERK1/2 levels were corrected by total ERK1/2 densitometry. * = p<0.01 vs WKY. **D:** To evaluate the effects of CaMKII inhibition on CaMKII subcellular compartmentalization, nuclear extract from WKY, SHR, SHR-AntCaNtide and SHR-tat-CN17β were prepared as indicated in methods. Nuclear extracts were analyzed by WB for total CaMKII with specific antibody. CaMKII levels were averaged and normalized to histone 3 densitometry.*, *P* < 0.05 *vs*. WKY. **E:** The nuclear extract from WKY, SHR, SHR-AntCaNtide and SHR-tat-CN17β were analyzed by WB for total ERK with specific antibody to test the effects of CaMKII inhibitors on ERK subcellular compartmentalization. CaMKII levels were averaged and normalized to histone 3 densitometry.*, *P* < 0.05 *vs*. WKY. **F**: To examine the association between CaMKII and ERK in the left ventricle from SHR following intramyocardial injections, total cell lysate from WKY, SHR, SHR-AntCaNtide and SHR-tat-CN17β, was immunoprecipitated using anti-CaMKII antibody and subjected to WB with anti-ERK antibody. ERK levels were averaged and normalized to IgG densitometry. *P < 0.05 vs. WKY. Data from all immunoblots presented here were quantified by densitometric analysis. Each data point in all graphs represent the mean±SEM of 3 independent experiments.

To confirm the role of the cross talk between CaMKII and ERK in cardiac hypertrophy, we evaluated CaMKII and ERK phosphorylation and subcellular localization in left ventricles. We observed that CaMKII expression levels were increased regardless of treatment in SHR compared to WKY (Fig 8A). Importantly, CaMKII phosphorylation levels were enhanced, and this effect was prevented by AntCaNtide and tat-CN17β (Fig 8B). As expected, increased levels of ERK phosphorylation were observed in sham treated SHR left ventricles. Once again treatment with CaMKII selective inhibitors markedly reduced ERK1/2 phosphorylation levels (Fig 8C). In the hypertrophic hearts, the nuclear accumulation of CaMKII and ERK was also enhanced (Fig 8D and 8E), as well as the interaction between both kinases (Fig 8F). Strikingly, CaMKII inhibition by AntCaNtide or tat-CN17β blocked both ERK1/2 phosphorylation (Fig 8C) and CaMKII/ERK interaction (Fig 8F). These inhibitory events were mirrored by the reduction of the nuclear content of both kinases (Fig 8D and 8E). These data indicate that CaMKII inhibitors, AntCaNtide and tat-CN17β, are both able to reduce hypertrophy of cardiac myocytes and remodeling of the heart, by a mechanism that involves the crosstalk between the ERK and CaMKII pathways and their nuclear accumulation.

## Discussion

In the present study, we provide for the first time the compelling evidence that pharmacological selective inhibition of CaMKII results in the reduction of cardiac hypertrophy both *in vitro* and *in vivo* models. Indeed, so far the role of CaMKII in LVH was essentially demonstrated by gene deletion strategies (5). Here we demonstrate that peptides designed on the CaMtide sequence can inhibit CaMKII and block hypertrophy responses. Furthermore, our data indicate that a crosstalk between CaMKII and ERK also occurs in cardiac myocytes and promotes cardiac hypertrophy.

Our data exploit the pathogenic role of CaMKII in the settings of cardiac myocyte hypertrophy, showing that a targeting strategy based on selective peptides can prevent this maladaptive response of the heart. Since CaMKII is present in humans in 4 isoforms, namely CaMKIIα, CaMKIIβ,CaMKIIγ and CaMKIIδ and the adult heart is composed of ∼56% myocytes, 27% fibroblasts, 7% endothelial cells, and 10% vascular smooth muscle cells [46], we felt the urge to characterize the expression of CaMKII isoforms in our cardiac myocyte models, namely the H9C2 myoblasts and adult rat ventricular cardiomyocytes. Both H9C2 and adult rat cardiomyocytes express CaMKII α, β, γ and δ isoforms, and this finding is in accordance with previous literature[47–51], albeit other reports suggest that the heart does not express CaMKII α and β isoforms[52]. Of note, though, our results indicate that the most abundant CaMKII isoform in adult rat cardiomyocytes is CaMKII β, since CaMKII α, δ and γ are only detectable when concentrated by immunoprecipitation. These data contradict the notion that CaMKIIδ is the most important CaMKII isoform in the heart. By a critical perusal of the literature, it appears that this assumption is based on early reports that analyzed CaMKII expression by mRNA expression from mRNA extracted by the whole heart [10, 53–55]. On the contrary, we evaluated expression of the isoforms using commercial, validated antibodies which are of wide use by researcher in the field and that recognize in rat the respective isoforms of CaMKII. Furthermore, our results are obtained in cultured cardiac myoblasts or freshly isolated ventricular myocytes; we also compared the relative expression in these cells with the most abundant source of CaMKII isoforms in the body of the rat, the brain. Therefore, we are convinced that differences in the model used and dissimilarities between mRNA and proteins may account for the divergences observed between our and previous reports. Our data do not exclude that less expressed isoforms of CaMKII might play a role in LVH; on the contrary, we believe that our results point in particular to the nuclear isoforms of CaMKII. In this regard, we confirm that CaMKII δ is the most important nuclear isoform in cardiac myocytes.

Our data, furthermore, indicate that the recently described mechanism of stimulation of nuclear transcription by CaMKII activation of ERK is possibly relevant also in the setup of LVH. In particular, we demonstrate that inhibition of CaMKII results in the loss of ERK accumulation within the nucleus. These data are in agreement and help to reconcile previous literature. In particular, it has been observed that nuclear inhibition of CaMKII does not prevent hypertrophy in response to physiological stimulation [56]. On the contrary, another report shows that CaMKII isoforms deletion results in attenuation of hypertrophy [5, 57]. According to our results both findings can co-exists, since the crosstalk between ERK and CaMKII takes play in the cytosol and favors accumulation of ERK in the nucleus. Selective inhibition of nuclear CaMKII isoform will not prevent ERK to enter the nucleus and determine activation of ERK dependent transcription factors related to hypertrophy [58].

The mechanism of inhibition of CaMKII by CaMKII-KIIN-based peptides represents an alternative strategy based on the steric inhibition of a conformational change of the kinase that is needed for its activation, rather than with the occupation of the ATP pocket (21). Although previously used for proof of concept studies, these peptides, and in particular AntCaNtide, were never used before *in vivo*. CaMKII selective inhibition with both AntCaNtide and tat-CN17β efficiently reduced the hypertrophy of cardiac myocytes, as well as the remodeling of the heart. We therefore considered antCaNtide as a leading compound, and identified its minimal inhibitory sequence, that resides in residues 1–17 (27). The novel tat-CN17β peptide recapitulates the inhibitory properties of the parental AntCaNtide peptide, both *in vitro* and *in vivo*, and the shorter sequence provides a basis for the identification of the minimal inhibitory sequence and the future molecular design of pharmacological inhibitors. Our findings retain most relevance in light of the multiple signaling converging on CaMKII, in different cellular types that contribute to the development of LVH.

Neurohormonal signals elevated in heart disease can stimulate CaMKII activity. Of interest, β-adrenergic[59] and Angiotensin II (7) receptor stimulation both independently result in CaMKII activation. Likewise, CaMKII inhibition reverses the hypertrophic and anti-apoptotic effects of these agonists, respectively [7, 60]. Also in inflammatory cells, CaMKII participates to AT1R-mediated NAD(P)H oxidase activation, leading to generation of reactive oxygen species, widely implicated in vascular inflammation and fibrosis. ANG II also promotes the association of scaffolding proteins, such as paxillin, talin, and p130C as, leading to focal adhesion and extracellular matrix formation [61]. All these phenotypes, being CaMK dependent, could be mitigated by a selective inhibitor of CaMK activity.

### Perspectives

Our data represent the first demonstration that pharmacological inhibition of CaMKII is effective in reducing cardiac myocyte hypertrophy in an animal model of hypertension-induced LVH. This study might provide the basis for a further exploration of the hypertension related phenotype in the SHR, such as cardiac arrhythmias, sudden death and survival. Also, it is a further advance in the understanding of physiology of cardiac remodeling that could be useful to develop targeted therapies in LVH models as well as other models of heart failure and impaired cardiac function
